# Environmental mobility barriers and walking for errands among older people who live alone vs. with others

**DOI:** 10.1186/1471-2458-13-1054

**Published:** 2013-11-08

**Authors:** Li-Tang Tsai, Merja Rantakokko, Erja Portegijs, Anne Viljanen, Milla Saajanaho, Johanna Eronen, Taina Rantanen

**Affiliations:** 1 Gerontology Research Center and Department of Health Sciences, University of Jyväskylä, P.O.Box 35, Jyväskylä, FI-40014, Finland

**Keywords:** Aging, Mobility, Environmental barriers, Living arrangements

## Abstract

**Background:**

Walking is the most popular form of physical activity among older people and for community-dwelling older people walking for errands is especially important. The aim of this study is to examine the association between self-reported environmental mobility barriers and amount of walking for errands among older people who live alone compared to those who live with others.

**Methods:**

This observational study is based on cross-sectional data on 657 people aged 75–81 living in Jyväskylä, Central Finland. Self-reports of environmental mobility barriers were collected under four categories: Traffic, Terrain, Distances and Entrance. Persons who reported walking for errands ≤ 1.5 km/week or at most once a week were categorized as having low amount of walking for errands (LOWER). High walking for errands (HIGWER) was defined as the highest quartile of kilometers walked per week (cut-off 8.5 km, referent). The rest were defined as having moderate amount of walking for errands (MODWER). Multinominal regression analysis was used to compare the odds for LOWER vs. HIGWER and MODWER vs. HIGWER, which were formed for each environmental mobility barrier separately.

**Results:**

Participants walked on average 6.5 km (SD 5.2) and 4.0 times (SD 2.2) per week and 14% reported LOWER. Persons living alone (57% of the participants) reported environmental mobility barriers more often than those living with others. LOWER was more common among those living with others. Among those living with others, all the environmental mobility barriers increased the odds for LOWER. In turn, among those living alone, only Distance- and Entrance- related environmental mobility barriers increased the odds for LOWER. People living alone typically run errands by themselves and become better aware of the barriers to environmental mobility, while those living with others have less exposure to environmental mobility barriers, as their walking for errands is more likely to be low.

**Conclusions:**

These findings emphasize the need to take living arrangements into account when analyzing the association between environmental mobility barriers and walking for errands. Future longitudinal studies are warranted to better understand the temporal order of events and to find ways to enhance walking for errands among older people.

## Background

Physical inactivity becomes more common with increasing age. Among older people, however, walking even short distances may help maintain health and functioning. In an American study, walking at least eight blocks per week helped maintain mobility in terms of walking speed [1]. For older people, walking is a feasible and popular form of physical activity [2] and ideal in the context of public health promotion [3]. Furthermore, engaging in community walking is an important contributory factor for social participation [4]. Walking for errands is a meaningful form of walking, which can be easily integrated into the daily routine of community-dwelling older people [5]. A German study found over eighty percent of community-dwelling older people run their errands on foot [6], and results from Finnish samples showed older women preferred walking to the use of cars or public transport for errands [7].

For older people, the home environment and its immediate surroundings may be a decisive factor for engaging in outdoor physical activity, especially among people who report mobility limitations [8]. Environmental mobility barriers increase unmet physical activity need (the feeling that one’s physical activity is inadequate) and fear of moving outdoors among older people [9,10]. Environmental facilitators for mobility such as having a park nearby decrease the risk for developing walking difficulty among older people [11]. Environmental features may be more closely associated with walking for errands than with walking for leisure among older people [12,13]. Walking for errands among older people is positively related to access to services and mixed land use [12,14]. Presence of multiple environmental mobility facilitators can motivate older people to walk for errands [15]. So far, there is limited information about the association between environmental mobility barriers and walking for errands. Further understanding of this topic is a crucial step towards promoting optimal mobility among older people, defined as being able to choose when, where, and how one wishes to go, safely and reliably [16].

A growing number of older people, mostly women, live alone in their own homes. Living alone may be associated with a higher likelihood of walking outside the home, as there is no other person to take care of the errands [17]. In another study, older people living with their families had better physical health status and more health-promoting behaviors than those living alone [18]. Understanding the dynamics between physical activity, environmental mobility barriers and living arrangements may help to detect need for support in physical activity participation among community-dwelling older people.

The aim of the present study was to examine the association between self-reported environmental mobility barriers and amount of walking for errands among older people with different living arrangements.

## Methods

The present study is based on cross-sectional analyses of the baseline data of the Screening and Counseling for Physical Activity and Mobility (SCAMOB) project, which is a randomized controlled trial on physical activity counseling (ISRCTN 07330512). The participants were community-dwelling people aged 75–81 years living in Jyväskylä in Central Finland. The urban area where the study was conducted is characterized by small hills. Many streets are rather quiet with predominantly only residential traffic with some streets with more traffic intersecting. There are several small parks with seating areas. Most of the shops and other services are concentrated in the city center which is located also in the center of the current study perimeter.

In accordance with the SCAMOB main goal, the inclusion criteria were: (1) the ability to walk at least 0.5 km without assistance, (2) at most moderately physically active, (3) no memory impairment, (4) no medical contraindications for physical activity and (5) informed consent [19,20]. The present analysis comprises data on the 657 community-dwelling people who took part in a home-based face-to-face interview and functional tests in the study center at baseline.

The ethical committee of the Central Finland Central Hospital approved the SCAMOB project. Participants were informed about the research before signing a consent form.

Walking for errands was elicited with the question “How much do you walk outdoors in the course of your daily activities, such as shopping, walking to the bus stop, etc.?” Participants were asked to report the average distance and frequency of their walking for errands during one week. We categorized walking for errands into three levels (low, moderate, and high amount) based on the following criteria: Low amount of walking for errands (LOWER) was defined as walking no more than 1.5 km/week or at most once a week, and has been found to be associated with elevated mortality and functional capacity decline among older people [21,22]. High amount of walking for errands (HIGWER) was defined according to the amount walked by those in the highest quartile of distance walked/week, which in our study population corresponded to more than 8.5 km/week. Those who did not fall into the above two categories were defined as having moderate amount of walking for errands (MODWER). The reliability for the categorization as assessed with Kendall’s tau-b was found to be good (r = 0.93) in a study among 29 older people interviewed two weeks apart [19]. Altogether 14 people (2% of the study population) had missing data on walking for errands.

Environmental mobility barriers were self-reported during an interview with standardized questions. Participants were asked whether a certain environmental mobility barrier hindered their possibility of moving outdoors independently (yes/no). Self-reported environmental mobility barriers to moving outdoors were categorized into four groups: Traffic (noisy traffic and dangerous crossroads), Terrain (hilly terrain and poor street condition), Distances (long distance to services and lack of resting places) [23] and Entrance (outdoor/indoor stairs present, no elevator, heavy doors, slippery floor and inadequate lighting). For the data analysis, environmental mobility barriers were dichotomized according to presence.

Living arrangements were self-reported during an interview according to four alternatives: living alone, living with a spouse, living with own child/children, and living with relatives. Only 2% of the participants lived with somebody other than a spouse and these individuals were included in the same category for the data analysis (dichotomized into living alone and living with others).

Sociodemographic indicators included age, gender, perceived financial status (very bad, bad, or moderate vs. good or very good), and years of education. Number of chronic conditions were first self-reported (physician-diagnosed chronic conditions lasting more than 3 months), and then further confirmed by the study nurse in a clinical examination. Use of a cane was self-reported. Depressive symptoms were assessed on the Center for Epidemiologic Studies Depression Scale (CES-D) [24]. Maximal walking speed was measured with a stopwatch over a distance of 10 m in the study center corridor. Participants wore suitable footwear for walking and used a walking aid if needed.

Participants’ characteristics were described using means and standard deviations (SD) or percentages according to amount of walking for errands and living arrangements, and differences were tested with chi-square tests for categorical variables and *ANOVA* or *t-test* for continuous variables. *T-test* was also used for analyzing differences in distance and frequency in walking for errands according to environmental mobility barriers.

We observed a significant interaction between living arrangements and environmental mobility barriers for the odds of low walking activity (p < 0.001). Two sets of multinominal regression analyses were performed to identify the associations between environmental mobility barriers and walking for errands. In the first set of analysis, participants were stratified according to their living arrangements (living alone or living with others). For each environmental mobility barrier the odds for LOWER and MODWER were computed separately with HIGWER used as the reference value.

In the second set of multinominal regression analysis, we included all the participants in the same analysis by creating a combined distribution for the independent variables. For the living arrangements, and for each environmental mobility barrier, the following categorization was computed: lives alone and reports a barrier, lives with others and reports a barrier, lives alone and does not report a barrier, and lives with others and does not report a barrier. As the reference group, we used those who lived alone and did not report a barrier, as they had the lowest prevalence of LOWER. The odds for LOWER and MODWER vs. HIGWER were calculated separately for each environmental mobility barrier by living status categorization.

All multinominal regression analyses were adjusted for age and gender. Owing to the low number of people in some categories of the independent variables, we added walking speed, number of chronic conditions, and CES-D score into the models one at a time to control for health differences (models not shown but data available from the authors upon request). Men and women were included in the same models, as gender-stratified analyses produced practically identical results. Results are reported as odds ratios (OR) and 95% confidence intervals (CI). Differences were considered to be statistically significant when p ≤ 0.05.

Statistical analyses were performed using the SPSS program (SPSS 19.0 for Windows/Mac, IBM).

## Results

The average age of the participants (n = 657) was 77.6 ± SD 1.9 and 75% were women. The mean self-reported weekly walking distance was 6.4 ± 5.1 kilometers and walking frequency was 4.0 ± 2.2. Individual and environmental characteristics are shown in Table 1, categorized according to low, moderate, and high amount of walking for errands as well as living alone vs. living with others. Distances as an environmental mobility barrier was associated with LOWER while the other environmental mobility barriers did not show a clear association with walking for errands. People who lived alone reported more environmental mobility barriers and were less often in the LOWER category than those living with others. HIGWER did not clearly differ between those living alone vs. living with others. Terrain was the most common environmental mobility barrier (33%), followed by Traffic (21%), Entrance (20%) and Distances (18%). Mean walking distances and frequency according to the presence of each environmental mobility barrier is shown in Table 2. Participants who reported Distances as a barrier walked fewer kilometers and less frequently than those who did not report Distances as a barrier.

**Table 1:** Characteristics of the participants according to amount of walking for errands and living arrangements

	**LOWER**	**MODWER**	**HIGWER**	p-value*	**Living alone**	**Living with others**	**p-value†**
	**n = 96**	**n = 381**	**n = 166**		**n = 381**	**n = 276**	
	**Mean ± SD**	**Mean ± SD**	**Mean ± SD**		**Mean ± SD**	**Mean ± SD**	
Age (years)	77.5 ± 2.0	77.6 ± 2.0	77.6 ± 1.9	0.875	77.8 ± 2.0	77.3 ± 1.9	0.002
Education (years)	8.9 ± 4.2	9.1 ± 4.0	9.3 ± 4.8	0.723	8.6 ± 4.1	9.8 ± 4.3	0.001
Chronic conditions (number)	3.8 ± 2.3	3.0 ± 1.9	2.7 ± 1.8	< 0.001	3.1 ± 2.0	2.9 ± 1.9	0.051
Walking speed (m/s)	1.2 ± 0.4	1.3 ± 0.4	1.5 ± 0.3	< 0.001	1.3 ± 0.3	1.4 ± 0.4	< 0.001
CES-D (score)	11.2 ± 8.2	10.5 ± 7.5	8.8 ± 7.2	0.020	10.4 ± 7.6	9.9 ± 7.6	0.375
Walking for errands							
Distance/week	1.2 ± 1.0	4.6 ± 1.8	13.5 ± 4.5	< 0.001	6.6 ± 4.5	6.2 ± 5.9	0.456
Frequency/week	1.4 ± 1.9	3.8 ± 1.7	5.9 ± 1.5	< 0.001	4.3 ± 2.0	3.6 ± 2.3	< 0.001
	%	%	%		%	%	
Female	56	83	69	< 0.001	90	54	< 0.001
Living alone	30	65	59	< 0.001			
Use of a cane (indoors or outdoors)	22	12	8	0.003	14	10	0.075
Perceived financial situation				0.604			< 0.001
Good or very good	40	41	45		35	50	
Very bad, bad or moderate	60	59	55		65	50	
Environmental mobility barriers							
Distances	31	18	8	< 0.001	21	15	0.049
Terrain	28	36	29	0.131	38	27	0.004
Traffic	19	22	19	0.542	22	20	0.534
Entrance	25	22	15	0.107	23	16	0.020
Amount of walking for errands							< 0.001
Low					8	24	
Moderate					85	66	
High					7	10	

*one-way ANOVA & Chi-square.

† t-t est & Chi-square.

*SD* Standard Deviation.

*CES-D* Center for Epidemiologic Studies Depression Scale.

*LOWER* Low amount of walking for errands, *MODWER* Moderate amount of walking for errands, *HIGWER* High amount of walking for errands.

NOTE: Environmental mobility barriers studied were Traffic (noisy traffic and dangerous crossroads), Terrain (hilly terrain and poor street condition), Distances (long distance to services and lack of resting places), and Entrance (outdoor stairs present, indoor stairs present, no elevator, heavy doors, slippery floor and inadequate lighting). LOWER: ≤ 1.5 km/week or at most once a week; HIGWER: ≥ 8.5 km/week (highest quartile); MODWER: those who did not fall into LOWER or HIGWER categories.

**Table 2:** Average distance and frequency walked in a week by participants reporting environmental mobility barriers

**Environmental mobility barriers**	**Distance (km) walked/week (n = 642)**	p-value*	**Frequency of walking/week (n = 649)**	p-value*
	**Mean ± SD**			**Mean ± SD**	
Traffic		0.675		0.179
Yes	6.3 ± 5.0		4.2 ± 2.2	
No	6.5 ± 5.2		3.9 ± 2.2	
Terrain		0.173		0.685
Yes	6.0 ± 5.1		4.0 ± 2.1	
No	6.6 ± 5.2		4.0 ± 2.2	
Distances		< 0.001		< 0.001
Yes	4.5 ± 4.4		3.1 ± 1.9	
No	6.8 ± 5.2		4.2 ± 2.2	
Entrance		0.120		0.469
Yes	5.8 ± 5.1		3.9 ± 2.1	
No	6.6 ± 5.1		4.0 ± 2.2	

* *t*-test.

NOTE: Environmental mobility barriers studied were Traffic (noisy traffic and dangerous crossroads), Terrain (hilly terrain and poor street condition), Distances (long distance to services and lack of resting places), and Entrance (outdoor stairs present, indoor stairs present, no elevator, heavy doors, slippery floor and inadequate lighting).

Table 3 presents the age and gender-adjusted odds for LOWER and MODWER with HIGWER as the reference. We observed a significant interaction between living arrangements and environmental mobility barriers for the odds of low walking activity (p < 0.001), and thus participants were stratified according to their living arrangements. Among people living alone, in general, the presence of an environmental mobility barrier increased the odds for LOWER, although not all the associations reached statistical significance. For those living alone and those living with others, Distances consistently increased the odds for LOWER.

**Table 3:** Odds ratios* for low and moderate amount of walking for errands with perceived environmental mobility barriers among those living alone and living with others

	**Living alone**		**Living with others**	
	**LOWER**	**MODWER**	**LOWER**	**MODWER**
	**(n = 29)**	**(n = 248)**	**(n = 67)**	**(n = 133)**
Environmental mobility barrier	OR (95% Cl)	OR (95% Cl)	OR (95% Cl)	OR (95% Cl)
Traffic	1.95 (0.76-5.01)	1.32 (0.72-2.40)	0.64 (0.25-1.64)	1.25 (0.59-2.62)
Terrain	1.83 (0.78-4.31)	1.38 (0.83-2.29)	0.75 (0.34-1.66)	1.12 (0.58-2.17)
Distances	7.77 (2.94-20.56)	1.93 (0.96-3.91)	7.35 (2.00-26.99)	2.86 (0.80-10.22)
Entrance	8.76 (3.37-22.80)	2.13 (1.09-4.20)	0.59 (0.22-1.57)	0.75 (0.34-1.64)

*OR* Odds Ratio, *CI* Confidence Interval.

*LOWER* Low amount of walking for errands, *MODWER* Moderate amount of walking for errands, *HIGWER* High amount of walking for errands.

* Adjusted for age and gender from multinominal logistic regression: LOWER and MODWER each compared to HIGWER. Models are computed separately for those living alone and those living with others.

Environmental mobility barriers studied were Traffic (noisy traffic and dangerous crossroads), Terrain (hilly terrain and poor street condition), Distances (long distance to services and lack of resting places), and Entrance (outdoor stairs present, indoor stairs present, no elevator, heavy doors, slippery floor and inadequate lighting). LOWER: ≤ 1.5 km/week or at most once a week; HIGWER: ≥ 8.5 km/week (highest quartile); MODWER: those who did not fall into LOWER or HIGWER categories.

Table 4 reports the multinominal regression models showing the associations between each of the four mutually exclusive environmental mobility barriers and amount of walking for errands categories. For each model, people living alone and not reporting the environmental mobility barrier were assigned as the reference group. In general, the presence of environmental mobility barriers increased the odds for LOWER (vs. HIGWER), with the majority of associations reaching statistical significance. For Traffic and Terrain, living with others and not reporting mobility barriers in these categories resulted in the highest odds for LOWER. Reporting Distances as a mobility barrier increased the odds for LOWER almost eight-fold among those living alone and more than thirty-fold among those living with others compared with the reference group.

**Table 4:** Odds rations* for low and moderate amount of walking for errands among older people with different living arrangements and perceived environmental mobility barriers

**Environmental mobility barrier**	**LOWER vs. HIGWER**			
	**Living alone (n = 29)**		**Living with others (n = 67)**	
	**n**	**OR (95% Cl)**	**n**	**OR (95% Cl)**
Traffic				
Yes	9	2.00 (0.78-5.10)	9	2.76 (1.00-7.59)
No	20	1.00	58	4.18 (2.16-8.08)
Terrain				
Yes	13	1.85 (0.79-4.33)	14	3.27 (1.33-8.07)
No	16	1.00	53	4.45 (2.19-9.03)
Distances				
Yes	14	7.55 (2.88-19.78)	16	30.45 (7.83-118.44)
No	15	1.00	51	4.18 (2.06-8.47)
Entrance				
Yes	16	8.82 (3.41-22.82)	8	3.98 (1.37-11.57)
No	13	1.00	59	6.97 (3.34-14.54)
	**MODWER vs. HIGWER**			
	**Living alone (n = 248)**		**Living with others (n = 133)**	
Traffic				
Yes	55	1.30 (0.71-2.35)	30	1.22 (0.59-2.54)
No	193	1.00	103	1.04 (0.66-1.64)
Terrain				
Yes	98	1.41 (0.85-2.33)	40	1.23 (0.64-2.34)
No	150	1.00	93	1.10 (0.68-1.79)
Distances				
Yes	51	1.98 (0.98-3.98)	17	2.78 (0.79-9.84)
No	197	1.00	116	1.03 (0.67-1.59)
Entrance				
Yes	59	2.15 (1.10-4.23)	23	0.94 (0.45-1.97)
No	189	1.00	110	1.21 (0.77-1.91)

*OR* Odds Ratio, *CI* Confidence Interval.

*LOWER* Low amount of walking for errands, *MODWER* Moderate amount of walking for errands, *HIGWER* High amount of walking for errands.

*Odds ratios adjusted for age and gender, calculated using multinomial logistic regression analyses comparing the odds of LOWER vs. HIGWER (upper panel) and MODWER vs. HIGWER (lower panel). Each environmental mobility barrier forms a separate model. Environmental mobility barriers studied were Traffic (noisy traffic and dangerous crossroads), Terrain (hilly terrain and poor street condition), Distances (long distance to services and lack of resting places), and Entrance (outdoor stairs present, indoor stairs present, no elevator, heavy doors, slippery floor and inadequate lighting). LOWER: ≤ 1.5 km/week or at most once a week; HIGWER: ≥ 8.5 km/week (highest quartile); MODWER: those who did not fall into LOWER or HIGWER categories.

Adding the number of chronic conditions and CES-D score into the models one at a time had no material influence on the odds ratios. Adding walking speed into the models attenuated the odds to some extent, but the pattern of associations remained similar to those in the models adjusted for age and gender (data not shown).

## Discussion

We observed that the association between self-reported environmental barriers and amount of walking for errands differed by living arrangements. People who lived alone were less likely to report LOWER but more likely to report environmental mobility barriers than those living with others.

Our findings may be explained in several ways. First of all, we focused only on walking for errands. It is likely that the need to run daily errands personally is greater when living alone than when living with others, which reduces the odds for LOWER. For people who do not live alone, their companions may run their errands for them, which may increase the risk for LOWER. Our findings are in line with two earlier observations, i.e. older people most often receive help from their spouse when their functional capacity declines [25] and that walking outside the home was more common for older women who live alone than those living with another person [17]. We speculate that, owing to their greater walking activity, those who live alone are more exposed to and hence better aware of the existing environmental mobility barriers, and consequently also more likely to report them. As those living with others have less experience of negotiating their nearby environment, they are probably also less aware of the environmental mobility barriers, which results in less reporting of them [26]. Reporting Distances as a mobility barrier was consistently associated with LOWER regardless of whether the person lived alone or with others. Distances as a mobility barrier increased the odds for LOWER to almost eight-fold for those living alone and more than thirty-fold for those living with others compared with the reference group. It is possible that people reporting Distances as a mobility barrier live in places where their destinations of interest are too far away to be reached on foot. Earlier studies have reported that older people who live close to particular destinations of interest to them walk more for transportation [12,27,28]. It is also possible that perceiving Distances as a mobility barrier reflects poorer health and physical functioning. This is supported by the fact that walking speed – an objective measure of physical functioning – attenuated the odds ratios to some extent. However, the odds ratios remained significant, and consequently we concluded that the association was not completely explained by poorer physical functioning among those reporting Distances as a mobility barrier.

Another interesting finding of our study was that Entrance as a mobility barrier was a stronger correlate of LOWER for people living alone than for those living with others. Entrance barriers may be easily overcome by the help of another person when exiting or entering the home. It is a reasonable assumption that people living alone more often need to negotiate Entrance barriers by themselves. Consequently, for people living alone, Entrance barriers may literally prevent them from going out.

Very low walking activity is a risk factor for further functional decline [1]. As people living alone had a lower prevalence of LOWER in our study, we suspect that older people living alone may be at a lower risk for functional decline in the future compared to those living with others. However, this needs to be confirmed in future studies. The present topic has not been widely studied previously. An earlier study showed comparable physical activity levels for those living with a spouse and those living alone [29]. Prospective studies showed that older women living alone had less functional decline and comparable risk for poor health outcomes than those living with a spouse [30,31]. However, we have not found earlier studies that have addressed the simultaneous associations of environmental mobility barriers and living arrangements with physical activity.

The main strength of this study is use of a large population-based sample to explore an increasingly topical issue [32-34]. Most of the participants were living in condominiums and some in detached houses within a radius of approximately 5 km in the same urban area. Hence the results are unlikely to be explained by differences in living environments. The age range is rather narrow, and consequently residual confounding due to age is not likely to explain the results either.

The study has some limitations. We studied only walking in the context of running errands. It is possible that some people were engaging in other important forms of walking, such as recreation or fitness, that were not included in the analysis. However, it is unlikely that a meaningful proportion of people would have been wrongly assigned to the LOWER category. Even if people have given up walking for fitness or leisure, walking for errands is maintained for as long as possible, as it allows people to maintain independent community living. Consequently, if people do not report walking for errands, it is highly likely that they are not active in other forms of walking either. An earlier longitudinal study found walking to be one of the most popular forms of customary physical activity among older people [35]. The present results are based on cross-sectional analyses, and consequently we cannot make inferences as to the temporal order of occurrence. It should also be noted that, stemming from the design of the original study, both the most active and the most disabled individuals were excluded. Therefore, the distribution of functioning and walking in the current study is located around the population average, but is likely to be somewhat truncated. Consequently, the present results are likely to underestimate the actual strength of the associations. However, we believe that similar associations would be found in any Western country.

## Conclusions

Among older people, the associations of environmental mobility barriers with walking for errands differ for those living alone vs. living with another person. Older people living with others had higher odds for low amount of walking for errands. This topic warrants further study to better understand the behavioral patterns that underlie the lower walking activity of these individuals and how they could be encouraged to be more physically active. Perceiving long distances to services as a mobility barrier was consistently associated with increased odds for low amount of walking for errands regardless of whether people lived alone or with others. Consequently, living in areas where there are local services and attractive walking destinations in the near vicinity of home may promote physical activity. The present results may be generalized to older people who are able to walk independently outdoors and who live in regular housing in urban areas. It is possible that some of the associations reported here may be due to poorer health among those who report environmental barriers and low amount of walking for errands. The present results may serve as a justification for future studies. Prospective studies would help to gain an idea of future trajectories of walking for errands, thereby clarifying the temporal order of the variables studied here, while qualitative studies would help us better understand the relevance of the studied associations in older people’s lives.

## Competing interests

The authors declare that they have no competing interests.

## Authors’ contributions

The authors are justifiably credited with authorship, according to the authorship criteria. LT: conception, design, analysis and interpretation of the data, writing the article; MR: conception, design, critical revision of the article; EP: conception, design, critical revision of the article; AV: conception, design, critical revision of the article. MS: conception, design, critical revision of the article; JE: conception, design, critical revision of the article; TR: conception, design, data collection, critical revision of the article, PI for the SCAMOB project. All the authors approved the final manuscript.

## Pre-publication history

The pre-publication history for this paper can be accessed here:

http://www.biomedcentral.com/1471-2458/13/1054/prepub

## References

[B1] Simonsick EM Guralnik JM Volpato S Balfour J Fried LP Just get out the door! Importance of walking outside the home for maintaining mobility: findings from the women’s health and aging study J Am Geriatr Soc 2005 53 2 198 203 10.1111/j.1532-5415.2005.53103.x 15673341

[B2] Wong CH Wong SF Pang WS Azizah MY Dass MJ Habitual walking and its correlation to better physical function: implications for prevention of physical disability in older persons J Gerontol A Biol Sci Med Sci 2003 58 6 M555 M560 10.1093/gerona/58.6.M555 12807928

[B3] Owen N Humpel N Leslie E Bauman A Sallis JF Understanding environmental influences on walking: review and research agenda Am J Prev Med 2004 27 1 67 76 10.1016/j.amepre.2004.03.006 15212778

[B4] Leyden KM Social capital and the built environment: the importance of walkable neighborhoods Am J Public Health 2003 93 9 1546 1551 10.2105/AJPH.93.9.1546 12948978 PMC1448008

[B5] Murtagh EM Murphy MH Boone-Heinonen J Walking–the first steps in cardiovascular disease prevention Curr Opin Cardiol 2010 25 5 490 496 20625280 10.1097/HCO.0b013e32833ce972 PMC3098122

[B6] Hinrichs T Trampisch U Burghaus I Endres HG Klaaßen-Mielke R Moschny A Platen P Correlates of sport participation among community-dwelling elderly people in Germany: a cross-sectional study Eur Rev Aging Phy Activ 2010 7 2 105 115 10.1007/s11556-010-0063-8

[B7] Siren A Hakamies-Blomqvist L Does gendered driving create gendered mobility? Community-related mobility in Finnish women and men aged 65 Transport Res Part F: Traffic Psychol Behav 2006 9 5 374 382 10.1016/j.trf.2006.06.010

[B8] Rasinaho M Hirvensalo M Leinonen R Lintunen T Rantanen T Motives for and barriers to physical activity among older adults with mobility limitations J Aging Phys Act 2007 15 1 90 102 17387231 10.1123/japa.15.1.90

[B9] Rantakokko M Mänty M Iwarsson S Törmäkangas T Leinonen R Heikkinen E Rantanen T Fear of moving outdoors and development of outdoor walking difficulty in older people J Am Geriatr Soc 2009 57 4 634 640 10.1111/j.1532-5415.2009.02180.x 19392955

[B10] Rantakokko M Iwarsson S Hirvensalo M Leinonen R Heikkinen E Rantanen T Unmet physical activity need in old age J Am Geriatr Soc 2010 58 4 707 712 10.1111/j.1532-5415.2010.02792.x 20398151

[B11] Eronen J von Bonsdorff M Rantakokko M Rantanen T Environmental facilitators for outdoor walking and development of walking difficulty in community-dwelling older adults Eur J Ageing 2013 doi:10.1007/s10433-013-0283-7 10.1007/s10433-013-0283-7 PMC5549184 28804315

[B12] Shigematsu R Sallis J Conway T Saelens B Frank L Cain K Chapman J King A Age differences in the relation of perceived neighborhood environment to walking Med Sci Sports Exerc 2009 41 2 314 321 10.1249/MSS.0b013e318185496c 19127195 PMC7164822

[B13] Shimura H Sugiyama T Winkler E Owen N High neighborhood walkability mitigates declines in middle-to-older aged adults’ walking for transport J Phys Act Health 2012 9 7 1004 1008 22971878 10.1123/jpah.9.7.1004

[B14] Frank L Kerr J Rosenberg D King A Healthy aging and where you live: community design relationships with physical activity and body weight in older Americans J Phys Act Health 2010 7 1 S82 S90 20440017 10.1123/jpah.7.s1.s82

[B15] Van Cauwenberg J Clarys P De Bourdeaudhuij I Van Holle V Verté D De Witte N De Donder L Buffel T Dury S Deforche B Older adults’ transportation walking: a cross-sectional study on the cumulative influence of physical environmental factors Int J Health Geographics 2013 12 1 37 10.1186/1476-072X-12-37 10.1186/1476-072X-12-37 PMC3765082 23945285

[B16] Satariano WA Guralnik JM Jackson RJ Marottoli RA Phelan EA Prohaska TR Mobility and aging: new directions for public health action Am J Public Health 2012 102 8 1508 1515 10.2105/AJPH.2011.300631 22698013 PMC3464831

[B17] Simonsick EM Guralnik JM Fried LP Who walks? Factors associated with walking behavior in disabled older women with and without self-reported walking difficulty J Am Geriatr Soc 1999 47 6 672 680 10366165 10.1111/j.1532-5415.1999.tb01588.x

[B18] Sok SR Yun EK A comparison of physical health status, self‒esteem, family support and health‒promoting behaviours between aged living alone and living with family in Korea J Clin Nurs 2011 20 11–12 1606 1612 21366741 10.1111/j.1365-2702.2010.03551.x

[B19] Leinonen R Heikkinen E Hirvensalo M Lintunen T Rasinaho M Sakari‒Rantala R Kallinen M Koski J Möttönen S Kannas S Huovinen P Rantanen T Customer‒oriented counseling for physical activity in older people: study protocol and selected baseline results of a randomized‒controlled trial (ISRCTN 07330512) Scand J Med Sci Sports 2007 17 2 156 164 17394477 10.1111/j.1600-0838.2006.00536.x

[B20] Mänty M Heinonen A Leinonen R Törmäkangas T Hirvensalo M Kallinen M Sakari R von Bonsdorff MB Heikkinen E Rantanen T Long-term effect of physical activity counseling on mobility limitation among older people: a randomized controlled study J Gerontol A Biol Sci Med Sci 2009 64 1 83 89 19164270 10.1093/gerona/gln029 PMC2691194

[B21] Shimada H Ishizaki T Kato M Morimoto A Tamate A Uchiyama Y Yasumura S How often and how far do frail elderly people need to go outdoors to maintain functional capacity? Arch Gerontol Geriatr 2010 50 2 140 146 10.1016/j.archger.2009.02.015 19356806

[B22] Hakim AA Petrovitch H Burchfiel CM Ross GW Rodriguez BL White LR Yano K Curb JD Abbott RD Effects of walking on mortality among nonsmoking retired men N Engl J Med 1998 338 2 94 99 10.1056/NEJM199801083380204 9420340

[B23] Rantakokko M Iwarsson S Mänty M Leinonen R Rantanen T Perceived barriers in the outdoor environment and development of walking difficulties in older people Age Ageing 2012 41 1 118 121 10.1093/ageing/afr136 22086965

[B24] Radloff LS The CES-D scale: a self-reported scale for research in the general population Applied Psychol Measurement 1977 1 385 401 10.1177/014662167700100306

[B25] Stoller EP Earl LL Help with activities of everyday life: Sources of support for the noninstitutionalized elderly Gerontologist 1983 23 1 64 70 10.1093/geront/23.1.64 6219923

[B26] Fänge A Iwarsson S Accessibility and usability in housing: Construct validity and implications for research and practice Disabil Rehabil 2003 25 23 1316 1325 10.1080/09638280310001616286 14617438

[B27] Nagel CL Carlson NE Bosworth M Michael YL The relation between neighborhood built environment and walking activity among older adults Am J Epidemiol 2008 168 4 461 468 10.1093/aje/kwn158 18567638 PMC2727277

[B28] Michael Y Beard T Choi D Farquhar S Carlson N Measuring the influence of built neighborhood environments on walking in older adults J Aging Phys Act 2006 14 3 302 312 17090807 10.1123/japa.14.3.302

[B29] Satariano WA Haight TJ Tager IB Living arrangements and participation in leisure-time physical activities in an older population J Aging Health 2002 14 4 427 451 10.1177/089826402237177 12391994

[B30] Sarwari A Fredman L Langenberg P Magaziner J Prospective study on the relation between living arrangement and change in functional health status of elderly women Am J Epidemiol 1998 147 4 370 378 10.1093/oxfordjournals.aje.a009459 9508104

[B31] Michael YL Berkman LF Colditz GA Kawachi I Living arrangements, social integration, and change in functional health status Am J Epidemiol 2001 153 2 123 131 10.1093/aje/153.2.123 11159156

[B32] Gómez LF Parra DC Buchner D Brownson RC Sarmiento OL Pinzón JD Ardila M Moreno J Serrato M Lobelo F Built environment attributes and walking patterns among the elderly population in Bogotá Am J Prev Med 2010 38 6 592 599 10.1016/j.amepre.2010.02.005 20494235

[B33] Kemperman A Timmerman H Influences of built environment on walking and cycling by latent segments of aging population Transport Res Record: J Transport Res Board 2009 2134 1 9 10.3141/2134-01

[B34] Ståhl A Carlsson G Hovbrandt P Iwarsson S “Let’s go for a walk!”: identification and prioritisation of accessibility and safety measures involving elderly people in a residential area Eur J Ageing 2008 5 3 265 273 10.1007/s10433-008-0091-7 PMC5546335 28798578

[B35] Armstrong GK Morgan K Stability and change in levels of habitual physical activity in later life Age Ageing 1998 27 suppl 3 17 23 10.1093/ageing/27.suppl_3.17 10408679

